# Interactive Digital Visualization Counseling for Lifestyle Change in Patients at Risk of Cardiovascular Diseases: Randomized Controlled Trial

**DOI:** 10.2196/83488

**Published:** 2026-01-22

**Authors:** Adrijana Svenšek, Mateja Lorber, Zalika Klemenc-Ketiš, Lucija Gosak, Dominika Muršec, Gregor Štiglic

**Affiliations:** 1 Faculty of Health Sciences, University of Maribor, Žitna ulica 15, Maribor, 2000, Slovenia, 386 2 30 04 762; 2 Primary Healthcare Research and Development Institute, Community Health Centre Ljubljana, Ljubljana, Slovenia; 3 Department of Family Medicine, Faculty of Medicine, University of Maribor, Maribor, Slovenia; 4 Department of Family Medicine, Faculty of Medicine, University of Ljubljana, Ljubljana, Slovenia; 5 Faculty of Electrical Engineering and Computer Science, University of Maribor, Maribor, Slovenia; 6 Usher Institute, University of Edinburgh, Edinburgh, United Kingdom

**Keywords:** biological age, cardiovascular disease, CGM, healthy lifestyle, Petal-X, RCT, visualization, continuous glucose monitoring, randomized controlled trial

## Abstract

**Background:**

Cardiovascular disease (CVD) remains the leading cause of death. Primary prevention relies heavily on health risk assessments and lifestyle changes, which can reduce long-term risk and mortality. Digital health offers an accessible and cost-effective approach to support prevention, enabling data sharing and visualization of key indicators such as blood pressure and glucose fluctuations. These visual insights may help patients better understand the effects of lifestyle changes and enhance communication with health care providers.

**Objective:**

This research aims to evaluate whether the use of CVD risk visualization (Petal-X) and continuous glucose monitoring (CGM), alone or in combination, is associated with lifestyle changes and the perception of person-centered care (PCC) among patients at increased risk of CVD.

**Methods:**

We conducted a 4-arm, single-blind, 2×2 factorial randomized controlled feasibility trial in primary care. A total of 119 participants were enrolled, of whom 101 completed the 6-month follow-up. Participants were randomized to 1 of 4 arms: (1) Petal-X CVD risk visualization+CGM; (2) CGM only; (3) Petal-X only; or (4) standard care with routine lifestyle counseling and no digital tools. CGM was used for 10 days in the CGM arms. Since this was a feasibility trial, no formal sample size calculation was performed. Primary outcomes are healthy lifestyle (Health Lifestyle and Personal Control Questionnaire [HLPCQ]) and perception of PCC (Person-Centered Practice Inventory—Service User [PCPI–SU]), and secondary outcomes (Systematic Coronary Risk Evaluation 2 [SCORE2], anthropometrics, and biological age) were assessed at baseline and 6 months. Descriptive statistics and Kruskal-Wallis tests (*K* independent samples) were used for analyses.

**Results:**

At baseline, mean SCORE2 values ranged from 3.84 (SD 2.08) in intervention group 3 to 4.87 (SD 2.61) in intervention group 1, with the control group having a mean value of 4.53 (SD 3.63). Regarding the assessment of a healthy lifestyle, the domain of daily routine had the highest baseline scores across all groups (eg, mean 19.24, SD 5.87 in intervention group 1), and these scores improved by the final evaluation, although there were no statistically significant differences (*P*=.42) in changes between the groups. The perception of PCC was rated highest across all groups in the domain of shared decision-making, with no statistically significant differences (*P*=.26) between the groups. Results indicated improvements in healthy lifestyle habits, but the impact of interventions on perceived changes remained insignificant.

**Conclusions:**

Healthy lifestyle and perceived PCC scores improved, although no statistically significant between-group differences were found. Risk visualization appears to be a key tool for increasing CVD awareness and strengthening patient involvement in care planning. Longer interventions with larger samples are needed to clarify these effects and optimize digital tools for lifestyle change.

## Introduction

Chronic diseases are long-lasting and usually progressive in nature and not transmissible between people. They represent a major global health burden, both in terms of mortality and health care costs. There are 4 main types of chronic disease: cardiovascular disease (CVD), cancer, chronic respiratory diseases (such as chronic obstructive pulmonary disease and asthma), and diabetes. These chronic diseases are responsible for most deaths worldwide [1,2]. Importantly, these diseases share common modifiable lifestyle risk factors such as poor diet, physical inactivity, tobacco use, and harmful alcohol consumption [2-4]. In 2021, they were estimated to cause 20.5 million deaths, approximately a third of deaths worldwide, which affected more than half a billion people [5-8]. The prevalence of CVD is rising due to increases in obesity, hypertension, type 2 diabetes mellitus, and physical inactivity [5,9-12]. Given that unhealthy lifestyles are estimated to contribute to around 60% of chronic disease risk [3,5,13,14], managing behavioral and environmental risk factors remains one of the most effective approaches to alleviating the burden of CVDs. Targeted lifestyle interventions have been shown to reduce CVD incidence and related mortality over the long term [15,16]. According to Visseren et al [17], lifestyle improvement should be the first line of intervention, including dietary changes (eg, reducing salt, saturated fats, and alcohol) and physical activity. The guidelines divide patients into risk categories so that prevention can be targeted. The groups are: I-very high risk of CVD (>40% in the next 10 y), II-high risk of CVD (20%‐40% in the next 10 y), III-moderate risk of CVD (10%‐20% in the next 10 y), IV-low risk of CVD (<10% in the next 10 y) [18]. However, many individuals at risk for CVD do not understand their personal risk or the potential benefits of lifestyle change. Moreover, they may underestimate the consequences of inaction, which underscores the importance of interventions that enhance awareness and promote sustained behavioral change [19-21].

In this context, digital health has emerged as a promising tool. Technological innovations are increasingly being used to deliver scalable interventions aimed at improving health behaviors [22]. Smartphones, which are now owned by approximately 6.9 billion people worldwide (86% of the global population), have become a platform for delivering digital health interventions [23]. Evidence suggests that digital health applications can support lifestyle change, improve glycemic control [24,25], lower blood pressure (BP) [26], and even reduce hospitalizations when integrated into cardiac rehabilitation programs [27,28]. Digital technologies also offer new ways of presenting health information in an engaging and understandable way. Visual representations of risk (graphs, dendrograms, and interactive charts) can improve patients’ understanding of their condition and motivate behavior change [8,29-31]. As noted by Visseren et al [17], absolute risk and risk reduction are more easily understood when presented visually, rather than numerically. Advanced tools such as decision support systems and continuous glucose monitoring (CGM) devices provide real-time, personalized insights into patient health. These technologies support prevention and treatment by integrating clinical data with evidence-based knowledge to enable personalized interventions [32]. For example, CGM systems not only help reduce the risk of hypoglycemia and hyperglycemia but also encourage behavior change by providing instant feedback on glucose levels [33-35]. However, the successful implementation of digital health tools depends on both patient engagement and health literacy [36]. Without the ability to understand and act on digital information, even the most advanced tools may have a limited effect. This is where person-centered care (PCC) becomes crucial. By involving patients as active partners in their own care and decisions, PCC has been shown to enhance outcomes across a range of conditions [37,38]. Research focuses on how risk for CVD can be effectively visualized using digital health tools, with a particular focus on CGM and its role in supporting lifestyle change. In line with the principles of PCC, the aim is to understand how these technologies can promote greater patient engagement and ultimately contribute to improved CVD outcomes. Simultaneously, the research recognizes the need for high-quality evaluations of the tools and visualization strategies used in clinical settings.

## Methods

The research used a quantitative, multiarm design with data collected through validated questionnaires and direct participant measurements to comprehensively assess behavioral changes as well as subjective and objective health outcomes.

### Study Design and Research Protocol

A quantitative research design was used to obtain measurable and generalizable data, which were collected using a combination of validated questionnaires and direct participant measurements, allowing a comprehensive assessment of both subjective experiences and objective health indicators. The trial followed a 4-arm 2×2 factorial design, and the research protocol was registered at ClinicalTrials.gov (NCT06226948). The primary objective was to compare changes in lifestyle behaviors across these 4 arms and to explore whether the combined use of CVD risk visualization and real-time CGM (intervention group 1) leads to greater improvements than standard care. Standard care consisted of routine clinical management at the primary care level, including regular check-ups with a family physician or nurse, brief lifestyle counseling (on diet, physical activity, smoking, and alcohol use) according to national guidelines, and access to general educational materials on CVD prevention. No additional digital tools or enhanced monitoring were provided to the control group.

The secondary objective was to compare changes in estimated CVD risk (SCORE2 [Systematic Coronary Risk Evaluation 2]) across the 4 groups. Additionally, we aimed to determine which lifestyle domains changed in patients at risk of CVD following exposure to CVD risk visualization, real-time CGM, or their combination.

### Type of Study

This research was designed as a single-blind, 4-arm randomized controlled trial (RCT) and was conducted from May to December 2024. Participants were informed only about the intervention they received and were not informed about the full set of study arms or the interventions delivered in other groups (limited disclosure). This RCT was designed as a pragmatic feasibility study embedded in routine primary care. A formal sample size or power calculation was not performed, as this study was designed as a pragmatic feasibility trial. The primary aim was to evaluate feasibility, usability, and behavioral trends rather than to test definitive effectiveness. Therefore, the sample size was determined based on practical feasibility considerations, including the number of eligible patients available in primary care and the logistical capacity for conducting clinical and laboratory assessments. As a result, the study may have been underpowered to detect statistically significant between-group differences.

Participants were randomly assigned to 4 study arms:

- Intervention group 1: Petal-X CVD risk visualization+CGM
- Intervention group 2: CGM only
- Intervention group 3: Petal-X visualization only
- Control group: standard care

Participants were randomized in a 1:1:1:1 ratio into 4 arms using a computer-generated allocation sequence (RAND function in Microsoft Excel). The randomization sequence was generated by the study researcher. The same researcher enrolled participants and assigned them to the trial arms according to the generated sequence. Given the pragmatic feasibility design, formal allocation concealment procedures were not implemented; the enrolling researcher was aware of the upcoming assignment. Participants without a compatible smartphone were randomized only between the 2 non-CGM arms (Petal-X only vs standard care).

The study followed the intention-to-treat principle, whereby all participants were analyzed within the groups to which they were originally assigned, regardless of whether they completed the intervention [39]. This approach preserved the integrity of randomization and reduced bias.

The trial and its reporting were in accordance with the CONSORT (Consolidated Standards of Reporting Trials) 2010 flowchart and the CONSORT extension for pilot and feasibility RCTs (see *Results*), and the corresponding checklist has been completed and submitted (Checklist 1).

### Assessment Instrument

The primary outcomes (healthy lifestyle and perception of PCC) were assessed using 2 validated instruments: the Health Lifestyle and Personal Control Questionnaire (HLPCQ) and the Person-Centered Practice Inventory—Service User (PCPI-SU).

The following validated instruments were used in the research.

The HLPCQ [40] was used to assess lifestyle and perceived personal control. The questionnaire included demographic data (sex, age, marital status, number of children, smoking status, and education and employment status) and anthropometric measures (body weight and height). The HLPCQ consists of 26 items grouped into 5 domains. We used a validated Slovenian version of the questionnaire [41]. In the Slovenian validation study conducted among 666 adults at risk of CVD, the total scale showed good internal consistency (Cronbach *α*=0.85), with subscale α coefficients ranging from 0.59 to 0.88, indicating acceptable to good reliability [41]. In the present feasibility trial, we therefore relied on these previously established psychometric properties and did not repeat internal consistency testing (eg, Cronbach α) in our sample. The main aim of this questionnaire was to identify and quantify lifestyle patterns that reflect lifestyle change as reflected in stress levels and internal control over health. All domains were significantly positively related to each other, suggesting that people who adopt healthy eating habits and avoid harmful diets also follow a daily routine of activities, engage in organized exercise, seek social support, and take care of their mental health. Higher scores indicate a better lifestyle.

The PCPI-SU [42] was used to assess perceptions of PCC. It consists of 20 items and is designed to capture individuals’ views on their perceptions of PCC. It covers 5 domains: working with the patient’s beliefs and values (4 items), shared decision making (5 items), genuine collaboration (4 items), compassionate presence (3 items), and holistic action (4 items). The questionnaire was a 5-point Likert scale. Higher scores indicated better perceptions of PCC. In a Slovenian study among 426 adults with noncommunicable diseases, the PCPI-SU demonstrated good internal consistency, with Cronbach *α*=0.82 for the total scale [43]. Consistent with our feasibility design and to avoid redundancy, we did not recalculate Cronbach α in the current sample and relied on these existing reliability estimates.

Participants were shown the Petal-X visualization [44] using a graphical tool that illustrated their current level of CVD risk in a visual representation. This tool was based on the SCORE2 prognostic model [45], which calculates an individual’s 10-year risk of developing CVD. We then modified the parameters to reflect target values and used the visualization again to show how an individual’s risk could potentially be reduced through lifestyle changes and adherence to the guidelines provided. The code of the Petal-X dashboard is publicly available on GitHub [46], and the English version of the dashboard as a web application is available on the internet [47]. The dashboard was developed using the Observable framework, with the Petal-X visualization [44] implemented using the Observable Plot system [48]. Petal-X was developed using a human-centered design process with iterative prototyping and expert review to ensure clarity and interpretability of the risk visualization [44]. In addition, the underlying surrogate model of SCORE2 was quantitatively validated and demonstrated high fidelity to the original SCORE2 equations, and a controlled experiment with 88 health care students showed that Petal-X supported more accurate interpretation of modifiable risk factor contributions than standard SCORE2 graphical score charts, without reducing perceived transparency, trust, or intention to use the tool [44]. Based on this prior evidence, we considered Petal-X to be of sufficient quality and validity for use as an intervention tool in this feasibility trial. It is based on the validated SCORE2 prognostic model.

Biological age is calculated from plasma GlycanAge analysis [49] and is based on the algorithm developed by Levine et al [50], which incorporates 9 blood biomarkers most strongly associated with mortality: mean corpuscular volume, C-reactive protein, albumin, blood glucose, alkaline phosphatase, red cell distribution width, creatinine, lymphocyte percentage, and chronological age; CGM data is collected using the Dexcom system G7 [51], allowing for real-time monitoring of blood glucose levels.

### Description of the Research Process

**Figure 1.**
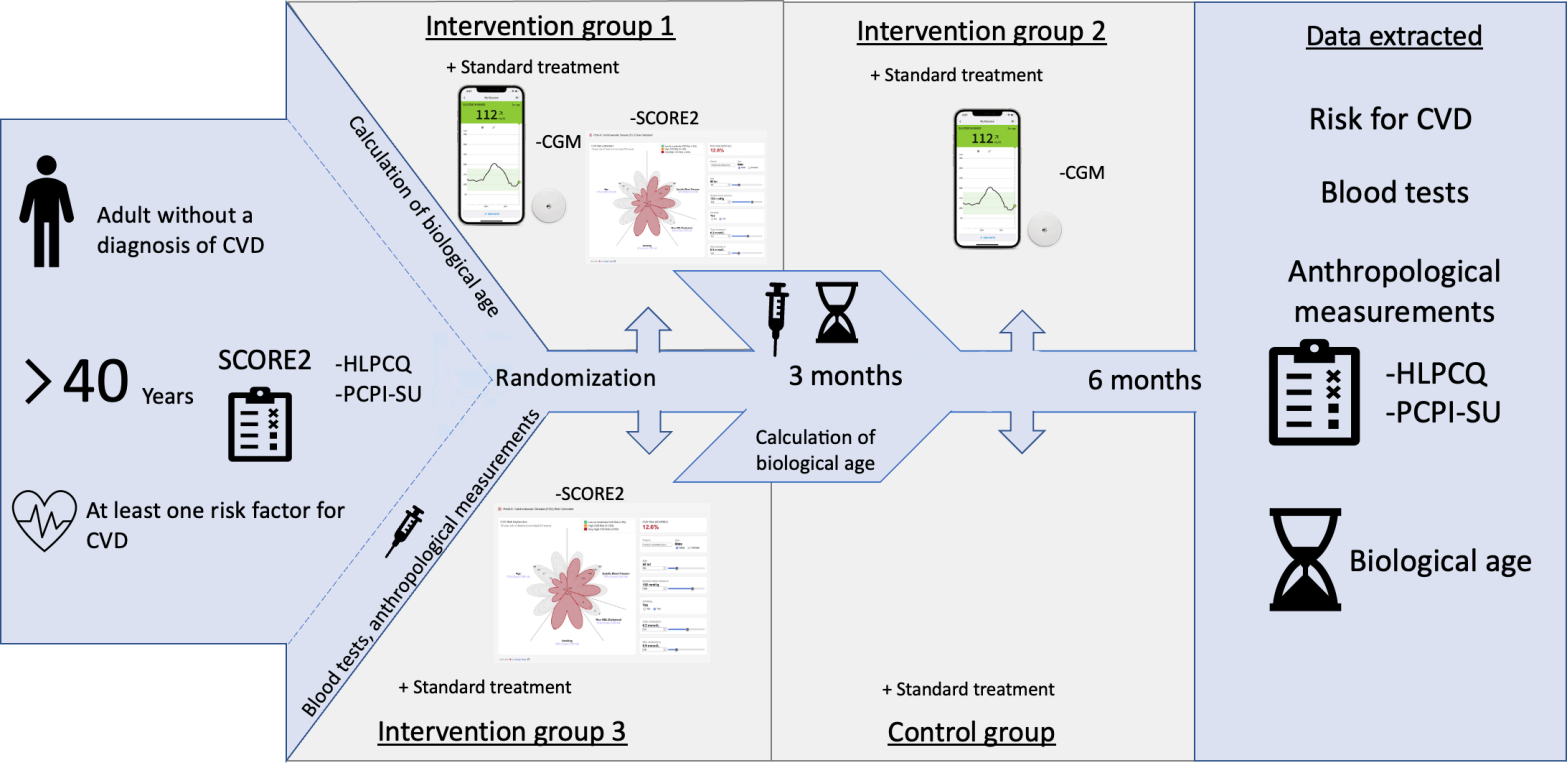
Intervention groups and research process. CGM: continuous glucose monitoring; CVD: cardiovascular disease; HLPCQ: Healthy Lifestyle and Personal Control; PCPI-SU: Person-Centered Practice Inventory—Service User; SCORE2: Systematic Coronary Risk Evaluation 2.

All participants who met the inclusion criteria completed the HLPCQ and the PCPI-SU at baseline and at the 6-month follow-up, and were also assessed for CVD risk using the SCORE2 prognostic model, incorporating the following variables: age, sex, systolic BP, total cholesterol, high-density lipoprotein (HDL) cholesterol, and smoking status. Venous blood samples were collected at baseline and at 6 months to determine standard biochemical markers (including lipid profile and fasting glucose) and to calculate phenotypic and glycated biological age, with an additional blood sample taken at 3 months for biological age assessment only. The risk assessment using SCORE2 was repeated after 6 months to evaluate potential changes. Additionally, biological age was calculated at 3 and 6 months as a more precise indicator of lifestyle changes compared to chronological age. Two models were used: the model by Levine et al [50], based on blood biomarkers, and GlycanAge, based on glycan analysis, as defined by Lauc and Primorac [49]. Participants were allocated to the following 3 different intervention groups and 1 control group according to the quantitative research design, with the groups differing according to the type of intervention they received during treatment (Figure 1).

Intervention group 1 received both CGM for 10 days and the Petal-X CVD risk visualization tool [44]. Petal-X allowed for a visual presentation of the participant’s current risk and an interactive simulation of how lifestyle changes could improve the risk profile.

Intervention group 2 used CGM for 10 days, without using the Petal-X CVD risk visualization tool.

Intervention group 3 received only the Petal-X risk visualization.

The control group received standard care without additional intervention and did not receive any of the digital interventions (Petal-X or CGM) after study completion, as the trial did not include a delayed-intervention phase.

At baseline and after 6 months, all participants underwent anthropometric and blood measurements, completed the HLPCQ and PCPI-SU questionnaires, and received updated CVD risk (SCORE2) and biological age assessments. These data allowed for comparisons between groups and evaluation of changes in lifestyle and CVD. The 6-month duration was selected based on previous studies demonstrating meaningful changes in health behaviors within this period [52,53]. Adverse events and device-related issues (eg, CGM-related problems) were monitored throughout the study. No serious adverse events were reported.

### Study Sample

Recruitment was open to individuals across Slovenia, but the promotional campaign and study implementation were concentrated in a Community Health Center in central Slovenia. Purposive sampling was used, and inclusion criteria included adults being 40 years or older with no previous diagnosis of CVD but with at least one of the following risk factors: elevated BP (systolic >140 mmHg), elevated fasting glucose (>6.1 mmol/L), abnormal lipid levels (total cholesterol >5 mmol/L, HDL <1.4 mmol/L for men and <1.6 mmol/L for women, low-density lipoprotein >3.5 mmol/L, triglycerides >1.7 mmol/L), or BMI >30.

Preexisting CVD, being younger than 40 years, and the absence of risk factors were the exclusion criteria. The sample was selected using purposive sampling. In total, 119 participants were included at baseline: 30 in intervention group 1 (CGM and Petal-X), 24 in intervention group 2 (CGM only), 33 in intervention group 3 (Petal-X only), and 32 in the control group. At follow-up, 101 participants remained: 27 in intervention group 1, 22 in intervention group 2, 26 in intervention group 3, and 26 in the control group. Dropout reasons included newly diagnosed health conditions, inability to attend follow-up appointments, or personal commitments.

### Statistical Analysis

Descriptive statistics (means and SDs for continuous variables and absolute and relative frequencies for categorical variables) were used to characterize the sample and summarize outcome measures. Group differences in categorical baseline characteristics (eg, gender, smoking status, and education) were analyzed using chi-square tests. All statistical analyses adhered to the intention-to-treat principle. IBM SPSS Statistics 29.0 was used for all analyses, and statistical significance was set at *P*≤.05.

For Hypothesis 1, differences in lifestyle change across the 4 randomized groups were analyzed using the Kruskal–Wallis test, with the HLPCQ total score as the dependent variable. For Hypothesis 2, changes in cardiovascular risk across the 4 groups were analyzed with SCORE2 as the dependent variable, also using the Kruskal–Wallis test, due to nonnormal distributions and modest cell sizes. HLPCQ and PCPI-SU scores were treated as continuous variables using their total scale scores.

Spearman’s rank correlation coefficients were calculated to explore associations between healthy lifestyle (HLPCQ) and perceived PCC (PCPI-SU) [54].

To reduce detection and analysis bias, outcome data were handled using coded group labels during data cleaning and analysis, and laboratory assessments were performed according to standard procedures without reference to group allocation.

### Ethical Considerations

All data collection and intervention procedures were conducted at a community health center in Central Slovenia. The research was carried out by a researcher under the supervision of an associate professor and a physician.

Participants underwent venous blood sampling at baseline, 3 months, and 6 months. Appointment invitations included a brief personalized summary of test results, which served to further motivate participants. The study was single-blind at the participant level: participants were informed only about procedures in their allocated arm and were not informed about the full set of study arms. Due to the pragmatic implementation, study personnel were not blinded.

The study was approved by the Ethics Committee for Nursing Research at the Faculty of Health Sciences, University of Maribor (Approval No. 02/7K-2023), and the National Medical Ethics Committee of the Republic of Slovenia (Approval No. 0120-217/2023/4). We have also registered the research protocol in clinical trials (ClinicalTrials.gov NCT06226948) [55]. All participants were informed that their data would be used exclusively for research purposes.

Participants received written and verbal information about the study and provided written informed consent before any study procedures. Participation was voluntary, and participants could withdraw at any time without consequences for their usual care. To protect privacy and confidentiality, each participant was assigned a unique study identification code; identifiable information was stored separately from research data and was accessible only to authorized study personnel. Data were handled in accordance with applicable data protection regulations (eg, General Data Protection Regulation) and institutional policies, and were stored on password-protected systems with restricted access; only anonymized or aggregated data were used for analysis and reporting. No financial compensation was provided for participation.

## Results

### Participant Enrollment

**Figure 2.**
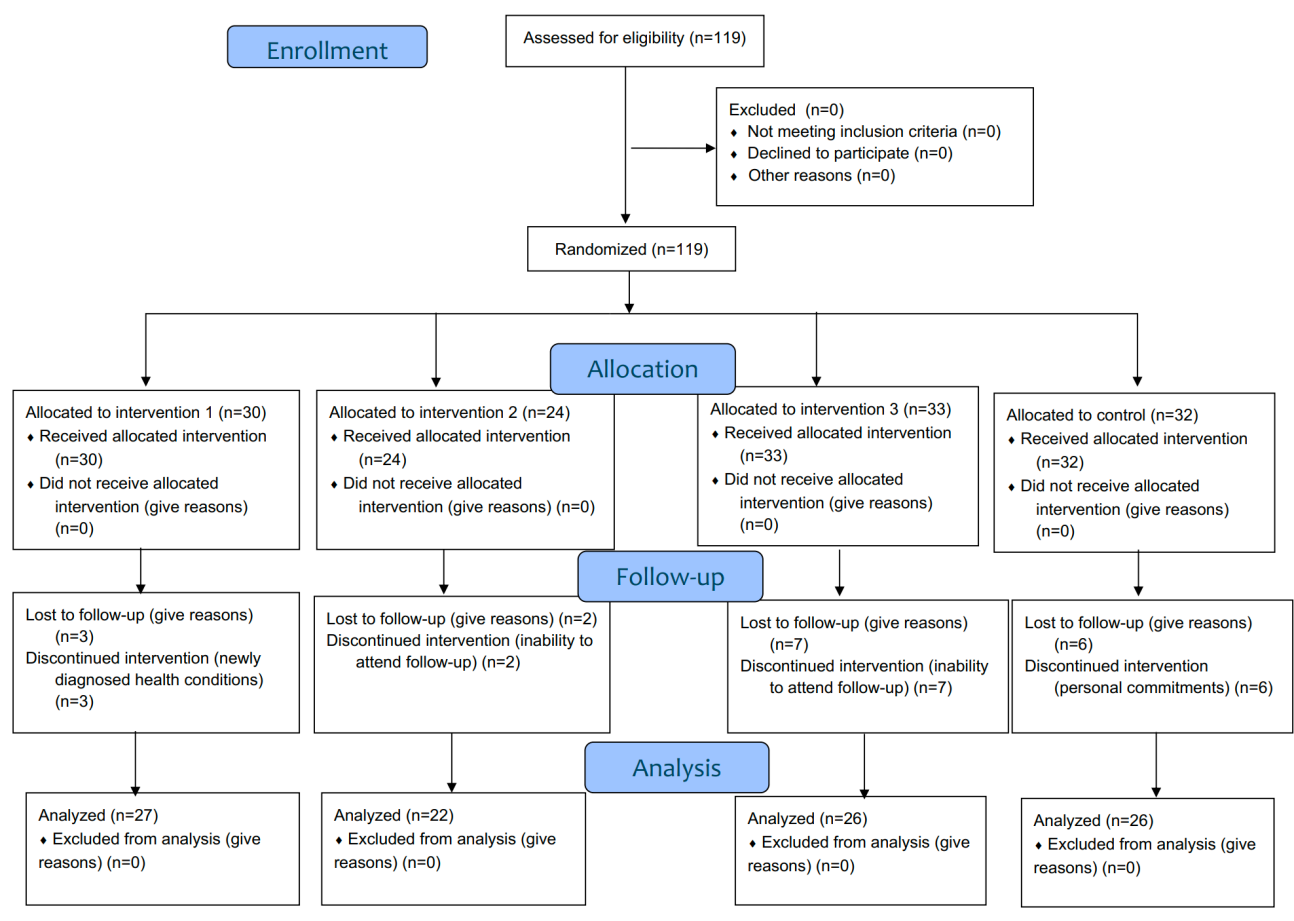
The CONSORT (Consolidated Standards of Reporting Trials) 2010 flowchart.

Figure 2 presents the CONSORT participant flow diagram. In total, 119 participants were enrolled and randomized into 4 arms, and 101 participants completed the 6-month follow-up; dropouts were mainly due to newly diagnosed health conditions, inability to attend follow-up appointments, or personal commitments.

### Demographic Characteristics

Of the 119 participants, 71% (n=85) were women and 29% (n=34) were men. At the start of the study, most participants were married (n=56, 47%) and had children (n=107, 90%). Most participants had specialization in a professional higher education program, completed a university program or a master’s degree (second degree; n=43, 36%), and were employed (n=110, 92%). Most participants did not smoke (n=82, 69%).

Table 1 shows the basic demographic characteristics of all intervention groups and the control group at baseline. The only demographic variable that differed significantly between the groups was educational level (*χ*²_15_=37.15; *P*=.001). Participants in Intervention group 1 (13/30, 43.3%) and Intervention group 3 (15/33, 45.5%) more frequently reported holding a master’s degree compared with the control group (6/32, 18.8%). No other demographic characteristics differed significantly across groups, including gender (*χ*²_3_=3.15; *P*=.37), age (H_3_=3.13; *P*=.37), marital status (*χ*²_12_=9.22; *P*=.68), having children (*χ*²_3_=0.71; *P*=.87), number of children (H_3_=0.94; *P*=.82), employment (*χ*²_6_=7.62, *P*=.27), smoking status (*χ*²_6_=2.08; *P*=.91), and number of cigarettes smoked (H_3_=3.18; *P*=.36). Any differences in the study were due to the interventions in the intervention groups and the level of education in the context of demographic characteristics.

**Table 1.** Descriptive characteristics of participants.

	Intervention group 1	Intervention group 2	Intervention group 3	Control group	*P* value
Gender, n (%)					.37
Man	9 (30)	9 (37.50)	6 (18.18)	11 (34.38)	
Woman	21 (70)	15 (62.50)	27 (81.82)	21 (65.63)	
Nonbinary	0 (0)	0 (0)	0 (0)	0 (0)	
Age (years), mean (SD)	51.87 (7.22)	48.50 (7.02)	50.33 (7.14)	49.84 (8.44)	.37
Marital status, n (%)					.68
Married	15 (50)	9 (37.50)	16 (48.48)	16 (50)	
Extramarital community	11 (36.67)	10 (41.67)	8 (24.24)	12 (37.50)	
Single	4 (13.33)	5 (20.83)	6 (18.18)	3 (9.38)	
Widower	0 (0)	0 (0)	2 (6.06)	1 (3.13)	
Other	0 (0)	0 (0)	1 (3.03)	0 (0)	
Having children, n (%)					.87
Yes	28 (93.33)	21 (87.50)	29 (87.88)	29 (90.63)	
No	2 (6.67)	3 (12.50)	4 (12.12)	3 (9.38)	
Number of children, mean (SD)	1.70 (0.84)	2.00 (1.22)	1.76 (1.03)	1.78 (1.01)	.82
Educational level, n (%)					.001
No education	0 (0)	0 (0)	0 (0)	0 (0)	
Primary education	1 (3.33)	0 (0)	0 (0)	0 (0)	
Secondary school education	8 (26.67)	7 (29.17)	2 (6.06)	13 (40.63)	
Higher university education	4 (13.33)	7 (29.17)	16 (48.48)	13 (40.63)	
Master’s degree	13 (43.33)	9 (37.50)	15 (45.45)	6 (18.75)	
PhD	4 (13.33)	0 (0)	0 (0)	0 (0)	
Other	0 (0)	1 (4.17)	0 (0)	0 (0)	
Employment, n (%)					.27
Yes	28 (93.33)	22 (91.67)	32 (96.97)	28 (87.50)	
No	2 (6.67)	0 (0)	1 (3.03)	1 (3.13)	
Retired	0 (0)	2 (8.33)	0 (0)	3 (9.38)	
Other	0 (0)	0 (0)	0 (0)	0 (0)	
Smoking status, n (%)					.91
Yes	6 (20)	5 (20.83)	8 (24.24)	8 (25)	
No	20 (66.67)	18 (75)	23 (69.70)	21 (65.63)	
Stop smoking	4 (13.33)	1 (4.17)	2 (6.06)	3 (9.38)	
Number of cigarettes, mean (SD)	15 (6.32)	9.80 (5.26)	11.50 (5.63)	9.75 (5.63)	.37

### Evaluation of the Measurements of the Participants in the Main Research

Intervention group 1 had the highest mean SCORE2 at baseline (4.87, SD 2.61), followed by the control group (4.53, SD 3.63) and intervention group 2 (4.08, SD 2.42), while intervention group 3 had the lowest score (3.84, SD 2.08). This indicates that intervention group 3 had the most favorable CVD risk profile at baseline, as lower scores reflect lower risk. Intervention group 1 had the highest mean total cholesterol value at baseline (5.93, SD 1.10), while intervention group 2 had the lowest mean value (4.82, SD 1.51). Intervention group 3 had the highest mean HDL value, with a final value of 1.62 (SD 0.34), while intervention group 2 had the lowest mean value, with a baseline value of 1.28 (SD 0.31).

The highest mean systolic BP was measured in intervention group 1, with a baseline value of 129.23 mmHg (SD 16.76), while the lowest mean was in intervention group 3, with a final value of 119.69 (SD 11.58).

There were no statistically significant differences between groups in SCORE2 (H_3_=3.269; *P*=.35), total cholesterol (H_3_=2.402; *P*=.49), HDL (H_3_=2.93; *P*=.40), systolic BP (H_3_=3.346; *P*=.34), diastolic BP (H_3_=2.588; *P*=.46), heart rate (H_3_=2.170; *P*=.54), BMI (H_3_=4.066; *P*=.25), phenotypic biological age (H_3_=2.708; *P*=.44) and glycated biological age (H_3_=0.261, *P*=.97). Statistical analysis showed no significant differences between groups. This means that the groups compared with respect to SCORE2, total cholesterol, HDL, BP, heart rate, BMI, phenotypic biological age, and glycated biological age were like each other. The baseline and final values for all measurements are presented in Table 2.

**Table 2.** Participant measurements by randomized groups.

	Intervention group 1, mean (SD)	Intervention group 2, mean (SD)	Intervention group 3, mean (SD)	Control group, mean (SD)	Change in *P* value (SD)
SCORE2^a^					.35
Baseline value	4.87 (2.61)	4.08 (2.42)	3.84 (2.08)	4.53 (3.63)	
Final value	4.06 (2.71)	3.53 (2.02)	3.17 (1.99)	4.36 (3.27)	
Total cholesterol					.49
Baseline value	5.93 (1.10)	5.09 (0.66)	5.68 (0.93)	5.28 (1.12)	
Final value	5.10 (2.26)	4.82 (1.51)	5.43 (0.99)	5.44 (1.07)	
HDL^b^					.02
Baseline value	1.43 (0.28)	1.28 (0.31)	1.61 (0.33)	1.33 (0.32)	
Final value	1.42 (0.31)	1.34 (0.31)	1.62 (0.34)	1.38 (0.32)	
Systolic blood pressure					.34
Baseline value	129.23 (16.76)	124.17 (14.21)	122.36 (13.32)	123.38 (17.09)	
Final value	128.26 (10.88)	121.36 (14.98)	119.69 (11.58)	124.77 (12.58)	
Diastolic blood pressure					.46
Baseline value	84.73 (8.73)	82.54 (11.92)	78.48 (9.86)	82.38 (10.93)	
Final value	78.93 (9.14)	77.18 (9.51)	75.35 (7.70)	78.19 (7.99)	
Heart rate					.54
Baseline value	76.07 (12.53)	73.79 (13.08)	72.15 (9.90)	71.66 (11.62)	
Final value	72.11 (8.38)	71.36 (12.69)	71.81 (9.33)	71.31 (9.70)	
BMI					.25
Baseline value	26.35 (4.19)	26.83 (4.50)	25.16 (4.03)	28.01 (5.64)	
Final value	26.48 (4.21)	26,55 (4.22)	24.48 (3.97)	27.65 (5.11)	
Phenotypic biological age					.44
Baseline value	46.82 (7.89)	43.30 (7.53)	43.90 (7.01)	43.34 (9.38)	
Final value	43.07 (14.02)	40.64 (11.36)	36.35 (16.98)	40.44 (16.43)	
Glycan biological age					.68
Baseline value	56.73 (16.17)	48.71 (21.73)	54.33 (16.08)	51.72 (18.31)	
Value after 3 mo	57.39 (16.53)	53.23 (20.79)	54.74 (16.00)	49.37 (19.8)	
Final value	56.04 (18.73)	51.86 (20.95)	54.96 (17.09)	53.50 (15.76)	

a SCORE2: Systematic Coronary Risk Evaluation 2.

b HDL: high-density lipoprotein cholesterol.

Based on the analyses of the data, we found that there were no significant differences in lifestyle changes between participants in the intervention groups and those in the control group.

### Healthy Lifestyle Assessment: HLPCQ

In all 3 intervention groups and the control group, the domain with the highest baseline score was daily routine. The mean scores for this domain were 19.24 (SD 5.87), 19.17 (SD 3.59), 19.93 (SD 5.18), and 17.61 (SD 5.82) for the first, second, and third intervention groups and the control group, respectively. However, the lowest-rated domain in all groups was organized exercise. The mean scores were 4.27 (SD 1.87), 4.00 (SD 1.78), 4.55 (SD 2.08), and 4.09 (SD 1.69).

At the end point, the highest and lowest rated domains remained unchanged but increased, indicating an improvement in HLPCQ scores. The mean scores for the highest-rated domain, Daily Routine, were: 21.27 (SD 4.94) for the intervention group 1; 21.50 (SD 4.30) for the intervention group 2; 21.25 (SD 3.90) for the intervention group 3; and 19.80 (SD 4.85) for the control group. However, organized exercise was the lowest rated domain in all groups. The mean scores were 5.31 (SD 1.67) in the first intervention group, 4.36 (SD 1.94) in the second intervention group, 4.54 (SD 2.00) in the third intervention group, and 4.88 (SD 2.01) in the control group.

Missing data were minimal across variables, with no missing values for healthy dietary choices at baseline (n=0), and only isolated cases for dietary harm avoidance (n=1), daily routine (n=3), organized physical activity (n=1), and social and mental balance (n=2); the highest number of missing values occurred in the total HLPCQ scores, with up to 5 in 1 group at final measurement.

There were no statistically significant differences in change in HLPCQ scores between groups (H_3_=2.801; *P*=.42). Thus, the Kruskal–Wallis test showed that group had no significant effect on the change in HLPCQ (Table 3).

**Table 3.** Baseline and final HLPCQ^a^ scores by randomized group.

HLPCQ	Intervention group 1, mean (SD)	Intervention group 2, mean (SD)	Intervention group 3, mean (SD)	Control group, mean (SD)
Healthy dietary choices				
Baseline value	15.93 (3.50)	14.79 (2.30)	16.94 (2.57)	15.25 (3.44)
Final value	17.72 (4.08)	16.64 (3.55)	18.28 (3.39)	16.42 (2.80)
Dietary harm avoidance				
Baseline value	10.40 (2.39)	10.52 (2.25)	11.31 (2.15)	10.28 (2.73)
Final value	11.08 (2.04)	10.32 (2.23)	11.88 (2.01)	10.46 (1.94)
Daily routine				
Baseline value	19.24 (5.87)	19.17 (3.59)	19.93 (5.18)	17.61 (5.82)
Final value	21.27 (4.94)	21.50 (4.30)	21.25 (3.90)	19.80 (4.85)
Organized physical activity				
Baseline value	4.27 (1.87)	4.00 (1.78)	4.55 (2.08)	4.09 (1.69)
Final value	5.31 (1.67)	4.36 (1.94)	4.54 (2.00)	4.88 (2.01)
Social and mental balance				
Baseline value	14.10 (2.51)	13.92 (2.54)	14.36 (2.36)	13.53 (3.29)
Final value	14.36 (2.04)	14.45 (2.82)	13.88 (3.05)	13.96 (2.73)
HLPCQ total				
Baseline value	64.17 (12.54)	63 (7.53)	66.76 (8.91)	60.58 (12.96)
Final value	69.50 (10.34)	67.27 (8.56)	68.90 (7.78)	65.72 (10.18)

a HLPCQ: Healthy Lifestyle and Personal Control Questionnaire.

The Kruskal–Wallis test (H_3_=2.801; *P*=.42) revealed no statistically significant differences between the groups regarding changes in HLPCQ. However, the control group showed the most significant improvement in terms of a healthy lifestyle.

### Perception of Person-Centered Care: PCPI-SU

At baseline, the highest-rated domain in all 3 intervention groups and the control group was sharing decision-making. The mean scores were 19.57 (SD 4.68) in the first intervention group, 18.00 (SD 4.64) in the second intervention group, 19.39 (SD 4.96) in the third intervention group, and 18.78 (SD 5.08) in the control group. However, engaging authentically was the lowest scoring domain in all groups, with average scores of 11.00 (SD 2.85) in the first intervention group, 10.29 (SD 2.48) in the second intervention group, 11.00 (SD 3.01) in the third intervention group, and 10.31 (SD 2.79) in the control group.

The domains that were highest and lowest rated at the final perception remained unchanged. The average domain scores for sharing decision-making in the first intervention group were 19.77 (SD 4.61), in the second intervention group 20.86 (SD 4.16), in the third intervention group 19.27 (SD 5.80), and in the control group 19.35 (SD 4.91). The lowest-rated domain was being sympathetically present in all groups. The average scores were 10.96 (SD 2.57) in the first intervention group, 11.55 (SD 2.99) in the second intervention group, 11.42 (SD 3.24) in the third intervention group, and 11.20 (SD 3.16) in the control group.

Missing data were low across variables, with no missing values for several baseline and final measurements. Occasional missing data were observed for working with the person’s beliefs and values (n=1), sharing decision-making (n=1), engaging authentically (n=3), being sympathetically present (n=1), and working holistically (n=2). The highest number of missing values occurred in the total PCPI-SU scores, with 3 at baseline and 2 at the final measurement (Table 4).

**Table 4.** The perception of PCPI-SU^a^ and components at the start and end of the study by the groups that were randomly selected.

	Intervention group 1, mean (SD)	Intervention group 2, mean (SD)	Intervention group 3, mean (SD)	Control group, mean (SD)
Working with the person’s beliefs and values				
Baseline value	15.43 (3.99)	14.00 (3.84)	15.15 (3.83)	14.44 (4.26)
Final value	15.23 (3.93)	15.27 (4.24)	14.88 (4.08)	15.35 (3.98)
Sharing decision-making				
Baseline value	19.57 (4.68)	18.00 (4.64)	19.39 (4.6)	18.78 (5.08)
Final value	19.77 (4.61)	20.86 (4.16)	19.27 (5.80)	19.35 (4.91)
Engaging authentically				
Baseline value	14.69 (3.41)	14.17 (3.24)	14.33 (3.37)	13.66 (3.64)
Final value	14.84 (3.14)	15.59 (3.26)	14.65 (3.50)	14.40 (3.69)
Being sympathetically present				
Baseline value	11.00 (2.85)	10.29 (2.48)	11.00 (3.01)	10.31 (2.79)
Final value	10.96 (2.57)	11.55 (2.99)	11.42 (3.24)	11.20 (3.16)
Working holistically				
Baseline value	14.55 (3.93)	13.61 (4.24)	14.42 (3.87)	13.87 (4.40)
Final value	14.23 (3.86)	15.00 (4.08)	15.50 (4.35)	14.77 (4.22)
PCPI-SU total				
Baseline value	75.64 (17.82)	70.13 (15.98)	76.70 (14.98)	71.17 (18.53)
Final value	74.92 (16.16)	79.19 (17.12)	75.73 (19.02)	73.54 (18.23)

a PCPI-SU: Person-Centered Practice Inventory—Service User.

This indicates that there are no significant differences between the groups in terms of changes in PCPI-SU. This is consistent with the results of the Kruskal–Wallis test, which showed no statistically significant differences (H_3_=4.056; *P*=.26). However, it shows that there was a difference in the change in the second intervention group, in which the CGM was worn. Based on this, it can be concluded that the intervention in the second group had an impact on improving the perception of PCC.

## Discussion

### Effectiveness of Risk Visualization and Glucose Monitoring in Reducing CVD Risk

The overall trend for the SCORE2 risk showed a decrease in CVD for all participants over the 6-month period. This suggests that participants showed some improvement in their risk profile regardless of the type of intervention. The group that was exposed to risk visualization through the Petal-X tool as part of the intervention showed the most improvement. When comparing all 3 intervention groups with the control group more explicitly, a clear pattern emerges. The group receiving combined CVD risk visualization and CGM showed the largest numerical reduction in SCORE2, followed by the group receiving visualization only, while the CGM-only group demonstrated a smaller improvement. The control group also exhibited a reduction in SCORE2, but the magnitude of change was consistently lower than in the visualization-based intervention groups. Although these differences did not reach statistical significance, the direction and relative size of the effects suggest that visual risk communication may have stronger motivational value than glucose monitoring alone, and that combining visualization with CGM may provide additional reinforcement. This highlights the potential value of visual feedback in supporting risk awareness and self-regulation. The findings are consistent with previous research indicating that traditional CVD factors, such as elevated BP, high cholesterol levels, and smoking, remain important in predicting future CVD events [56,57]. Despite the use of different methodologies and assessment tools in different studies, the consistency in core risk factors supports the reliability of tools such as SCORE2 for estimating individual risk. In the present study, the use of a validated prognostic model (SCORE2), combined with robust data collection from the Slovenian population, provided a solid foundation for evaluating risk trends.

Unlike other research designs, such as the focus group methods used by Vivek Dhukaram et al [58] or the large-scale registry analyses conducted in the Taiwanese study using the National Health Insurance Research Database [59], our approach provides clinically relevant insights into how risk evolves in real-world conditions. Furthermore, physician-led evaluations based on European guidelines, as demonstrated in the study by Dudas et al [60], continue to be a fundamental aspect of CVD risk management. Our findings support these approaches by integrating a PCC component through visualization, which may enhance patient engagement and understanding of personal risk. In line with international evidence [57,61], this study confirms that identifying at-risk individuals early on through structured risk assessments enables more precise preventive interventions. Visualizing risk, particularly when paired with ongoing monitoring or tailored counseling, may further encourage behavioral changes and reduce the long-term burden of CVD. The results of the CVD risk assessment allow for early identification of at-risk individuals, which may contribute to more targeted preventive interventions and a reduction of the burden of CVD in the population, and these findings are also consistent with the reviewed literature [56,57,61]. It is important to interpret the results within the context of this feasibility trial. The absence of statistical significance may be related to the modest sample size, baseline variability, and the short duration of exposure to the interventions. These factors limit the ability to detect smaller but potentially meaningful behavioral changes. Therefore, the results should be interpreted as preliminary indicators of trends rather than definitive effect estimates.

### Evaluating and Modifying Lifestyle Behaviors in CVD Risk Patients Through Visualization and Glucose Monitoring

Assessment of lifestyle using the HLPCQ revealed improvements in multiple domains, particularly among participants who received CVD risk visualization and CGM. These tools were most effective in promoting healthy dietary choices and enhancing daily routines, emphasizing the importance of combining risk awareness with real-time feedback [62]. Participants who received only visualization or only CGM also showed progress, though less consistently. Organized physical activity remained the weakest area across all groups, suggesting that general interventions may be insufficient to influence this behavior. Further research confirms the importance of routine, nutrition, and physical activity in improving quality of life and reducing the risk of disease [63-68]. Social and mental balance showed minimal change, which suggests that these aspects may be more resistant to digital or informational interventions and more dependent on the broader psychosocial context. The avoidance of dietary harm also decreased, particularly in groups without combined support. Older participants tended to report better organization of their lifestyle and healthier eating habits. This aligns with findings that age can positively influence adherence to preventive behaviors. Although no statistically significant differences were found, there were meaningful behavioral shifts in groups with combined interventions. The HLPCQ proved to be a useful tool for detecting these changes, particularly given its ability to incorporate self-regulation and health-related behaviors. Its future use in primary care and epidemiological research appears promising. It is important to note that lifestyle change depends not only on information but also on health literacy, motivation, and contextual support [69]. Research from Sweden has emphasized the importance of valid methods for engaging communities in sustainable prevention efforts, particularly among populations with low health literacy or limited access to care [70]. Therefore, enhancing health literacy could be central to national strategies for preventing lifestyle-related conditions [71]. In conclusion, visualization and CGM can support behavioral changes in individuals at risk of CVD, particularly regarding dietary and routine habits. However, more targeted approaches are needed for physical activity and psychosocial domains, which remain less responsive.

### Lifestyle Change Across Intervention and Control Groups

We tested the hypothesis that there would be significant differences in lifestyle change between groups, but this was not confirmed. The Kruskal–Wallis analysis revealed no statistically significant differences in HLPCQ score changes between the 4 study groups (3 intervention groups and the control group). The control group reported the greatest overall change in healthy lifestyle behavior, followed by smaller positive changes in all intervention groups. This suggests that the inclusion of new technologies, such as CVD risk visualization and CGM, did not result in statistically superior lifestyle improvements over 6 months. Nevertheless, the presence of positive trends in all intervention groups indicates a potential motivational effect of the interventions. A sustainable lifestyle change is a long-term process requiring continuity, reinforcement, and individual adaptation to have a lasting impact on quality of life and health-related well-being [17]. Based on other studies with similar intervention durations [17,53], we expected to detect measurable changes after 6 months. However, the short-term use of CGM and limited intervention exposure may have reduced the observable impact within the timeframe of our research.

Additionally, the Petal-X visualization of CVD risk was described as a concrete and motivating element that helped individuals recognize the necessary behavioral changes in their daily lives. These observations indicate that while measurable outcomes were modest, experiential factors may still play an important role in behavior change. Further research should explore longer intervention periods and enhanced support structures to evaluate the full potential of visual and self-monitoring tools in promoting healthy lifestyles. It is important to note that educational level differed significantly between the randomized groups at baseline. This imbalance is a common limitation in small feasibility samples, where randomization may not fully equalize sociodemographic characteristics. Since higher educational attainment is associated with better health literacy, greater engagement in preventive behaviors, and improved ability to interpret risk information, it is possible that educational level influenced participants’ responses to the interventions. Although we cannot determine the extent of this influence, it may have contributed to behavioral differences observed across groups. Future studies should include stratified randomization or larger sample sizes to minimize such baseline imbalances.

### Determining Whether Person-Centered Care Is Associated With Lifestyle Change

A link between PCC and lifestyle change was demonstrated. The results of the correlation analysis revealed a weak, yet statistically significant, positive relationship between perceived PCC and healthy lifestyle behaviors, including self-regulation. The Spearman correlation coefficient showed that higher HLPCQ scores, reflecting healthier lifestyle patterns and greater personal control, were associated with higher perceptions of PCC. Although the correlation was weak, its statistical significance suggests that the association is unlikely to be due to chance. These findings show that improving lifestyle and self-management behaviors could lead to a more positive perception of care quality from the patient’s perspective. Therefore, even minor improvements in perceived PCC could be meaningful, particularly when promoting long-term behavioral change and better health outcomes. Researchers emphasize the importance of health care professionals assessing individual risk, designing care plans that are tailored to patients’ lifestyles and contexts, and implementing PCC approaches to positively influence health outcomes [72-76]. Furthermore, interventions targeting lifestyle changes have been shown to improve BP and lipid profile control, highlighting the effectiveness of PCC in managing risk factors and encouraging behavioral changes [77]. This evidence highlights the importance of tailoring health care delivery to patients’ individual needs and preferences. PCC has the potential to improve patient satisfaction and may reduce CVD risk factors such as hypertension [78-80]. Therefore, integrating lifestyle-oriented PCC strategies into clinical practice should be considered a priority in both prevention and chronic care management.

These findings should be interpreted with caution, as the study may have been underpowered and the intervention period relatively short, both of which could have limited the ability to detect statistically significant effects. It is also important to consider potential clinical significance. Both the HLPCQ and PCPI-SU scores showed improvements across groups, including the control group. Although these changes were not statistically significant, small improvements in lifestyle behaviors or person-centeredness may still be clinically meaningful in a primary care setting. Behavior change is often gradual and incremental, and even minor positive shifts can contribute to long-term cardiovascular risk reduction when sustained over time.

### Limitations

This study has several limitations. The lack of a double-blind design may have introduced bias, and participants may have perceived differences between the intervention groups, which could have influenced the outcomes. The sample included only individuals with an increased risk of CVD based on clinical markers, excluding those with behavioral risk factors. Variability in digital literacy, particularly among older participants, may have affected uptake of the technology-supported interventions. Motivation fluctuated due to personal life events, and some technical issues with CGM devices occurred, but these were resolved. Although the lifestyle questionnaire had not been validated in Slovenia, the necessary cultural adaptations were made in line with WHO guidelines.

Additionally, the study was not sufficiently powered to detect small or moderate effects, as the sample size was limited and no formal power calculation was conducted prior to recruitment. This may have contributed to the absence of statistically significant findings despite observable clinical trends. The 6-month intervention period may have been too short to capture measurable changes in long-term lifestyle behaviors or CVD risk, which typically require sustained support and longer follow-up. This likely reduced the detectable impact of both visualization and CGM-based interventions. Consequently, the trial was not adequately powered to detect small or moderate between-group differences, and the absence of statistically significant findings should therefore be interpreted with caution.

### Key Findings and Recommendations

Although the quantitative results did not confirm statistically significant improvements, participants in the intervention groups reported enhanced awareness, motivation, and engagement in their health management. Digital tools such as Petal-X, CGM, and biological age assessment supported PCC and encouraged meaningful lifestyle adjustments. These findings demonstrate the potential for integrating digital tools into preventive health care to improve understanding of CVD risk and promote behavior change. Future studies should include larger and more diverse samples, extend the follow-up period, and examine effects in specific subgroups. Strengthening digital literacy among health care professionals and patients will be essential for successful implementation. With better training and user-friendly tools, technologies such as Petal-X and CGM could provide substantial support for lifestyle change, ongoing risk monitoring, and more effective PCC.

### Conclusions

This study makes a valuable contribution to the growing field of digital health, demonstrating the potential of combining risk visualization (Petal-X), CGM, and biological age assessment for the lifestyle-based prevention of CVD. While no clinically significant improvements were observed, the findings provide valuable insights into the feasibility, usability, and motivational impact of digital tools in PCC. Integrating such technologies could facilitate the early identification of risk, encourage patient engagement, and promote behavior change. These results emphasize the necessity of further longitudinal research and the development of digital infrastructure to facilitate lifestyle interventions within preventive health care in Slovenia. We also provided a detailed description of the standard care received by the control group to ensure transparency and comparability between intervention arms, in accordance with CONSORT recommendations.

This study highlights the potential of integrating digital tools such as Petal-X visualization, CGM, and biological age assessment into preventive health care. While statistical significance was limited, participants reported increased awareness, motivation, and involvement in managing their health. These findings support the value of PCC approaches and the need for further research on long-term, technology-supported lifestyle interventions. Expanding digital literacy and developing national guidelines for digital integration will be key to enhancing care quality and reducing CVD risk in the future.

## Supplementary material

10.2196/83488 Checklist 1 CONSORT-eHEALTH checklist (V 1.6.1).

## Abbreviations

BP: blood pressure
CGM: continuous glucose monitoring
CONSORT: Consolidated Standards of Reporting Trials
CVD: cardiovascular disease
HDL: high-density lipoprotein
HLPCQ: Healthy Lifestyle and Personal Control Questionnaire
PCC: person-centered care
PCPI-SU: Person-Centered Practice Inventory—Service User
RCT: randomized controlled trial
SCORE2: Systematic Coronary Risk Evaluation 2
